# Artificial intelligence–enabled multi-omics biomarkers for immune checkpoint blockade: mechanisms, predictive modeling, and clinical translation

**DOI:** 10.3389/fimmu.2026.1732079

**Published:** 2026-02-23

**Authors:** Xiaodong Wang, Di Xiong, Songli Cui, Bingchen Duan, Gouping Ding, Yiping Huang, Qianqian Wang

**Affiliations:** 1 Department of Oncology, Zhuzhou Hospital Affiliated to Xiangya School of Medicine, Central South University, Zhuzhou, China; 2 Department of General Medicine, Zhuzhou Hospital Affiliated to Xiangya School of Medicine, Central South University, Zhuzhou, China; 3 Department of Orthopaedic Surgery, Zhuzhou Hospital Affiliated to Xiangya School of Medicine, Central South University, Zhuzhou, China

**Keywords:** artificial intelligence, biomarkers, cancer, immunotherapy, multi-omics

## Abstract

Immune checkpoint inhibitors (ICIs) have transformed oncology, yet durable benefit remains confined to a minority of patients, revealing the limitations of single biomarkers such as PD-L1 expression, tumor mutational burden, and microsatellite instability. Multi-omics profiling, spanning genomics, transcriptomics, epigenomics, proteomics, metabolomics, microbiomics, and imaging-derived radiomics/pathomics, enables a systems-level interrogation of tumor–immune interactions. It captures lineage plasticity, antigen-presentation defects, metabolic and epigenetic suppression, stromal remodeling, and microbiome-driven immune tone that collectively shape ICI sensitivity and resistance. Artificial intelligence (AI) and machine learning are increasingly indispensable for fusing these heterogeneous, high-dimensional data into deployable composite predictors and mechanistically grounded signatures, while explainability approaches (e.g., SHAP, Grad-CAM) help link model outputs to actionable biology. This review synthesizes emerging AI-enabled multi-omics biomarkers across major tumor types, highlights clinical applications in response stratification, combination-therapy selection, and longitudinal monitoring, and discusses key translational barriers, including cohort and platform heterogeneity, limited prospective validation, privacy constraints, model drift, and equity. We conclude by outlining future directions in single-cell and spatial multi-omics integration, federated learning, and generative modeling to accelerate robust, generalizable precision immunotherapy. Pragmatic implementation will require harmonized pre-analytics, clinically feasible assays or distilled panels, and decision-support interfaces that communicate calibrated uncertainty to oncologists.

## Graphical Abstract

**Figure:**
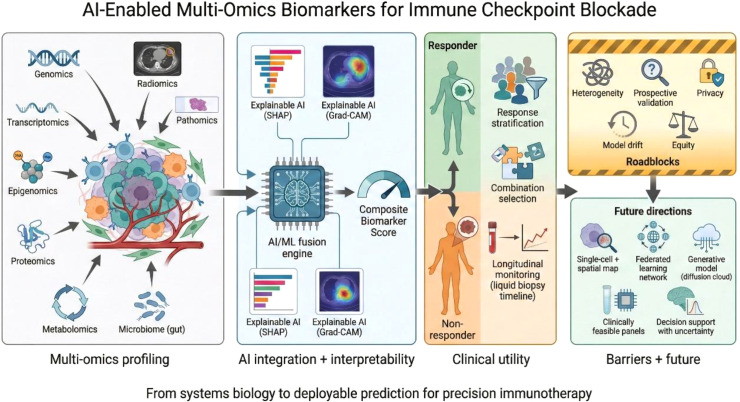

## 1 Introduction

Immune checkpoint inhibitors (ICIs), including antibodies targeting PD-1/PD-L1 or CTLA-4, have reshaped oncology by reinvigorating anti-tumor T cell activity and producing durable remissions in a subset of patients. Nevertheless, clinical benefit remains limited across many major malignancies, with overall response rates typically ~20–40% (1–3). Primary and acquired resistance are driven by tumor heterogeneity, immune-evasion programs, and dynamic tumor–microenvironment interactions (4). Accordingly, conventional biomarkers such as tumor PD-L1 expression, high tumor mutational burden (TMB), and microsatellite instability often provide incomplete or inconsistent prediction of benefit, motivating integrative strategies that better capture the multifactorial biology governing ICI efficacy (5, 6).

Multi-omics profiling offers a panoramic characterization of tumors across genomic, transcriptomic, proteomic, epigenomic, metabolomic, and microbiomic layers, and can be extended to imaging-derived “omics,” including radiomics from radiological scans and pathomics from digital histology. Integrated analyses clarify diverse routes of immune escape. Genomic studies have linked TP53 and CREBBP alterations with ICI non-response in diffuse large B-cell lymphoma, whereas transcriptomic signatures in responders frequently reflect activated immune pathways (7–9). Single-cell RNA sequencing in non–small cell lung cancer (NSCLC) further reveals lineage plasticity, with tumor cells adopting alternative differentiation states through SOX2/WNT/YAP-associated programs that foster an immunosuppressive microenvironment and resistance (10, 11). Epigenomic reprogramming also contributes; in nasopharyngeal carcinoma, Epstein–Barr virus–driven enhancer activation can induce genes such as CACNA2D1, promoting stem-like, immune-evasive phenotypes (12, 13). Metabolic rewiring intersects with immune suppression as well: increased lactate production and histone lactylation can blunt anti-tumor immunity, linking tumor metabolism to epigenetic dampening of immune responses (14, 15).

**Figure 1:**
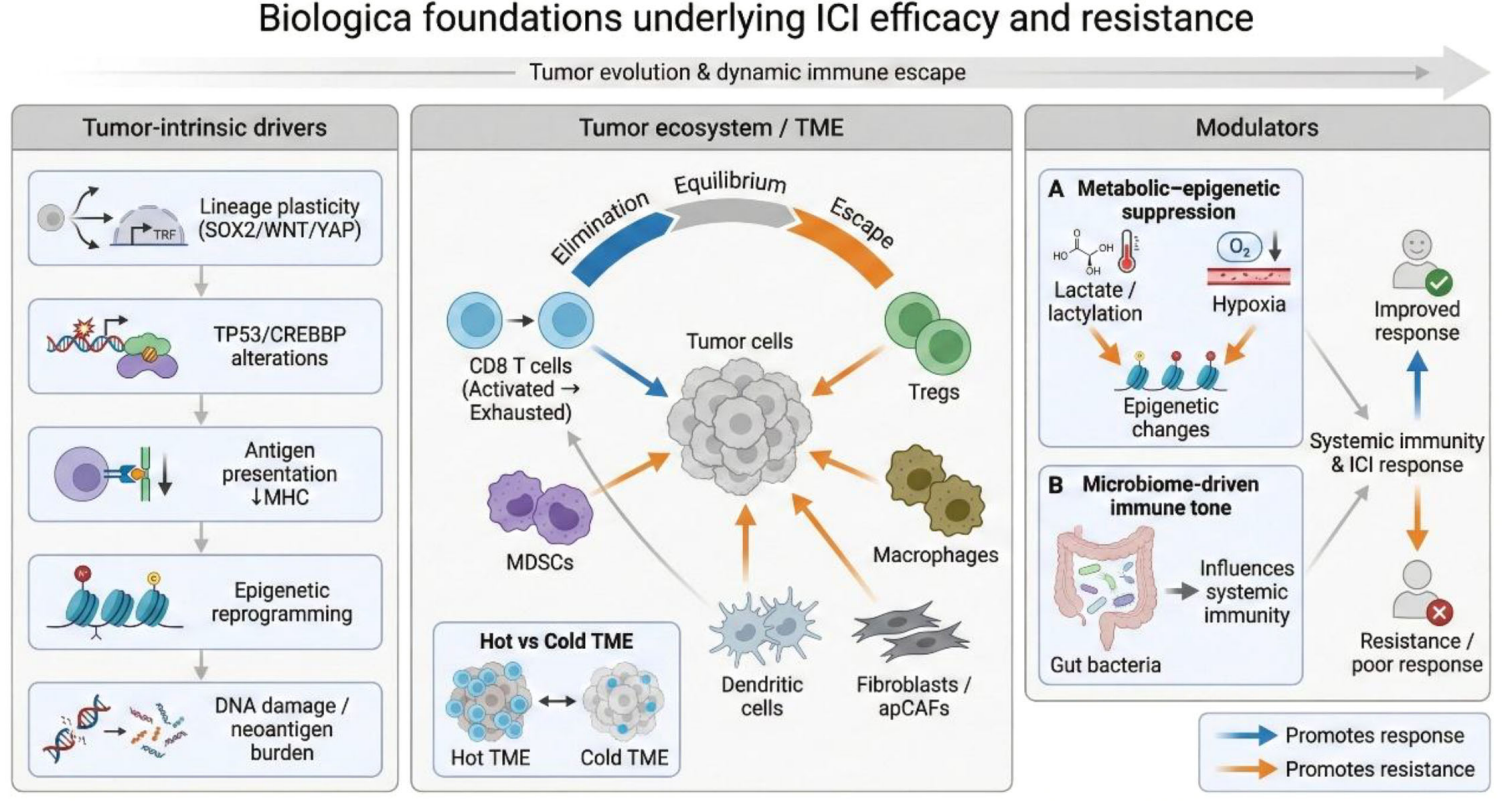
Biological foundations underlying ICI efficacy and resistance. Schematic overview of multilevel determinants shaping immune checkpoint inhibitor (ICI) response versus resistance across tumor evolution and dynamic immune escape. Tumor-intrinsic drivers include lineage plasticity (SOX2/WNT/YAP programs), TP53/CREBBP alterations, impaired antigen presentation (↓MHC), epigenetic reprogramming, and DNA damage/neoantigen burden. Within the tumor ecosystem/tumor microenvironment (TME), immunoediting states (elimination–equilibrium–escape) are influenced by CD8 T-cell activation/exhaustion and suppressive/structural components (Tregs, MDSCs, macrophages, dendritic cells, fibroblasts/apCAFs), contributing to “hot” versus “cold” TMEs. Systemic modulators include metabolic–epigenetic suppression (lactate/lactylation, hypoxia-driven epigenetic changes) and microbiome-driven immune tone. Blue arrows denote processes promoting response; orange arrows denote processes promoting resistance.

The gut microbiome adds another determinant of ICI efficacy, as microbial composition and metabolites influence systemic immunity. Differences between responders and non-responders are repeatedly observed; in NSCLC, enrichment of taxa including *Bacteroides* species has been associated with improved outcomes, whereas antibiotic-associated disruption of the microbiota correlates with poorer responses (16–18) (**Figure 1**).

Artificial intelligence (AI), spanning machine learning (ML) and deep learning (DL), is increasingly essential for distilling these high-dimensional, heterogeneous datasets into actionable composite biomarkers. Pathomics exemplifies this potential: DL models applied to colorectal cancer histopathology can link morphology to underlying genomic and transcriptomic states and accurately predict microsatellite instability (19, 20). In radiology, convolutional neural networks applied to NSCLC CT imaging extract radiomic features that, when combined with inflammatory indices, can stratify ICI responses (21, 22). AI has also been applied to metagenomic profiles; in NSCLC, random forest models identify gut microbial species such as *Bacteroides caccae* and *Prevotella copri* associated with favorable outcomes, illustrating how multi-modal integration can outperform single-modality predictors (23, 24).

Importantly, interpretability tools such as SHAP and Grad-CAM connect predictions to biology, strengthening mechanistic insight and clinical trust. In melanoma, LASSO-regularized integration of tumor exome and RNA-seq data outperforms TMB alone and highlights patient-specific genomic and transcriptomic drivers; imaging models similarly localize regions associated with benefit (24–26). Recent studies underscore translational promise: integrated genomic–transcriptomic–radiomic models improve prediction of anti–PD-1 outcomes in NSCLC (21, 24); AI-assisted liquid-biopsy analyses in hepatocellular carcinoma enable risk stratification and longitudinal monitoring of evolving resistance (27, 28); and urine-based methylation–mutation integration in bladder cancer distinguishes luminal versus basal subtypes to inform ICI use (29, 30). This review synthesizes these advances, discusses translational barriers, and outlines future directions including single-cell and spatial omics integration, federated learning for privacy and cohort diversity, generative modeling for data augmentation, and deeper incorporation of the microbiome–immune axis with rigorous validation and equity-focused development.

## 2 Biological foundations of ICI response and resistance

Immune checkpoint inhibitor (ICI) outcomes arise from a dynamic interplay between tumor-intrinsic programs and the immune contexture of the tumor microenvironment (TME). Tumors must both evade immune detection and resist cytotoxic attack, and multi-omics studies across cancers have clarified how genetic, epigenetic, and microenvironmental adaptations converge to prevent immune eradication (24, 26, 31). Resistance commonly reflects coupled tumor cell–intrinsic alterations and extrinsic constraints in the TME, where malignant, immune, and stromal compartments continuously interact and co-evolve (32, 33). These interactions are intrinsically dynamic: immune pressure can sculpt tumors by selecting immune-evasive clones, while tumor-derived signals reshape immune recruitment and function, yielding an evolving ecosystem that often favors suppression (34, 35).

A central mechanism of resistance is reduced antigenicity or impaired antigen presentation in the setting of an immunosuppressive microenvironment (36, 37). ICIs act by blocking inhibitory checkpoints such as PD-1/PD-L1, thereby releasing T-cell activity; however, if tumors downregulate antigen presentation or if T cells are rendered dysfunctional, checkpoint blockade has limited efficacy (36, 37). Lineage plasticity, the capacity of cancer cells to change differentiation state, is increasingly recognized as a driver of immune escape (10, 11, 38). In non–small cell lung cancer (NSCLC), single-cell analyses trace many tumors to an alveolar type II cell origin and show that a subset can transdifferentiate through SOX2/WNT/YAP signaling into more primitive states that resist T-cell killing (10, 11, 38). This lineage switching remodels the TME: tumor-derived factors recruit fibroblasts and macrophages that generate immunosuppressive cues, physically exclude cytotoxic T lymphocytes, and promote T-cell exhaustion (10). Such reciprocal tumor–stroma–immune interactions give rise to “cold” tumors characterized by sparse T-cell infiltration and abundant suppressive myeloid cells, which respond poorly to ICIs (10, 11, 39). Notably, resistant NSCLC TMEs can contain emergent subsets such as CXCL13^+^ exhausted CD8^+^ T cells and antigen-presenting cancer-associated fibroblasts, implying that even immune-edited tumors may retain exploitable pockets of antitumor potential if suppressive stromal crosstalk is disrupted (10, 11, 39).

The tumor–microbiome axis adds a further layer of complexity. Commensal bacteria, particularly in the gut, can systemically modulate immune tone. Fecal microbiota transplantation (FMT) studies suggest that transferring stool from an ICI-responsive donor can, in some cases, convert a previously resistant recipient into a responder, highlighting a causal contribution of microbial communities (40, 41). Mechanistically, a diverse microbiome may promote pro-inflammatory immunity and enhance T-cell priming. In NSCLC, metagenomic sequencing has linked taxa including *Bacteroides* and *Prevotella* species to improved anti–PD-1 outcomes, whereas antibiotic exposure, through perturbation of gut flora, has been associated with inferior efficacy. This may reflect elimination of beneficial microbes and altered microbial metabolism (17, 18). Importantly, not all microbiome changes are beneficial: FMT may also introduce organisms that generate immunosuppressive metabolites (16, 17). Thus, a “favorable” microbiome may augment systemic immunity (for example via enhanced Th1-associated responses or dendritic-cell activation), whereas an “unfavorable” microbiome may foster regulatory T cells or myeloid-derived suppressors that blunt immunotherapy (16, 17). Defining actionable microbial features remains an active area, with AI approaches increasingly used to discriminate beneficial from deleterious microbial signatures.

Cancer type–specific escape routes further shape ICI response. In renal cell carcinoma (RCC), bulk and single-cell transcriptomic analyses across large cohorts emphasize the role of HLA variation and myeloid–T cell interplay (31, 36, 42). Responding RCC tumors often exhibit an immunologically “hot” TME with pro-inflammatory M1-like macrophages and clonally expanded, interferon-γ–producing T cells recognizing neoantigens presented by particular HLA class I alleles (31, 36, 42). Non-responders more commonly show immunosuppressive M2 macrophages and T cells expressing inhibitory receptors, together with tumor cells that have lost immunogenic antigen expression or present suboptimal peptide repertoires, in part due to HLA alleles that skew presentation away from key tumor antigens (31, 36, 42). Machine learning applied to genomic data has supported composite “immune HLA scores” integrating HLA genotypes and neoantigen features to stratify RCC patients likely to benefit from checkpoint blockade, illustrating how subtle variation in antigen presentation capacity can tip the balance between rejection and escape (31, 36).

Multiple genomic alterations contribute to ICI resistance across cancers. Loss-of-function mutations in interferon-γ pathway components or antigen presentation genes such as B2M and JAK1/2 can drive primary resistance by impairing effective T-cell recognition (24, 37). Broader genomic analyses implicate tumor suppressors and stress-response regulators such as PTEN, KEAP1/NFE2L2, CREBBP, and TP53 in poor outcomes within specific contexts (32, 33). In gastrointestinal cancers treated with anti–PD-1, TP53 and CREBBP mutations are enriched in non-responders, plausibly by promoting an immune-cold phenotype through altered cytokine programs or reduced immunogenicity, often alongside heightened proliferation and immunosuppressive gene expression (32, 43). Importantly, genomic effects are context dependent: TP53-associated non-response has been reported predominantly in tumors with low PD-L1 expression, suggesting that when baseline immune activation is weak, TP53-mutant biology further biases the system toward escape (9, 32, 43). These observations reinforce that genomic predictors are tightly coupled to microenvironmental state and should be interpreted within integrated multi-omic frameworks.

Epigenetic reprogramming in tumor and immune compartments provides an additional mechanism of immune evasion. Global DNA hypomethylation and locus-specific hypermethylation can silence genes needed for immune recognition, including antigen presentation machinery and chemokines that recruit effector T cells (14, 44). Post-translational histone modifications integrate metabolic and signaling states with immune function (14, 44). A striking example is histone lactylation, an epigenetic mark derived from lactate abundant in hypoxic tumor regions. Tumor-exported lactate can enter immune cells and be converted to acetyl-CoA, promoting histone lactylation in tumor-infiltrating macrophages and skewing them toward an M2 immunosuppressive phenotype (14, 15, 45, 46). Elevated lactylation has been linked to radioresistance and ICI resistance in preclinical models, whereas inhibiting lactate production or uptake can reduce these suppressive modifications and restore immune activity (14, 15). Enhancer reprogramming represents another epigenetic route: in virus-associated nasopharyngeal carcinoma, Epstein–Barr virus can activate host enhancers that aberrantly induce oncogenes such as CACNA2D1, promoting a stem-like, immune-resistant state (12, 13). Consequently, epigenetic therapies are being explored as immunotherapy adjuncts to re-open silenced immune pathways and increase antigenicity.

A unifying concept underlying these mechanisms is tumor heterogeneity. Tumors comprise mosaics of subclones embedded in supportive stroma, enabling adaptation to immune attack: even if most cancer cells are eliminated, rare variants capable of immune evasion may survive and expand. Stem-like tumor populations are particularly resilient and often less immunogenic, for example by expressing lower MHC levels or higher checkpoint ligands (47–49). Across tumor types, high stemness, quantified by indices such as mRNAsi or CytoTRACE, associates with immune-cold TMEs, reduced CD8^+^ T-cell infiltration, and poor ICI outcomes (47, 49). Single-cell and spatial studies have implicated proliferative tumor states, enriched for chaperonins such as CCT5, as highly stem-like programs that correlate with sparse T-cell infiltration and inferior response (48, 49). Spatial transcriptomics further indicates that CCT5-high tumor clusters can be physically shielded from immune cells, and CCT5-enriched tumors show lower response rates to checkpoint blockade (48, 49). Similarly, in lung adenocarcinoma, proliferative progenitor states marked by genes such as MKI67 and STMN1 contribute to intratumoral heterogeneity and are linked to ICI failure (10, 11, 24). An AI-based stemness model stratifies lung adenocarcinoma into subgroups, with high-stemness tumors displaying markedly fewer tumor-infiltrating lymphocytes and minimal benefit from ICIs; multiplex immunofluorescence validation shows scarce CD8^+^ T cells in these high-stemness lesions (47, 49).

Collectively, tumors employ diverse, interacting evasion strategies, including lineage switching, antigenic editing, metabolic and epigenetic suppression, microbiome-mediated immune modulation, and stemness-driven heterogeneity, that determine ICI success or failure. Multi-omics studies, increasingly coupled with AI analytics, are disentangling these mechanisms and assembling resistance-associated biomarkers across cancers(**Table 1**). These insights argue that effective prediction of immunotherapy response will require integrating genetics, epigenetics, TME composition, and external determinants such as the microbiome rather than relying on any single parameter, providing the rationale for multi-modal biomarker development and AI-enabled integration in subsequent sections.

**Table 1:** Key mechanisms driving immunotherapy response/resistance revealed by multi-omics studies across cancers.

Cancer type	Key multi-omics mechanisms/findings linked to ICI response/resistance	Omics layers leveraged	Representative biomarkers/indicators	References
NSCLC	Spatially organized CAF programs associate with immune exclusion and immunosuppression; CAF–T cell crosstalk can induce CXCL13-related states linked to TLS biology and ICI outcomes	scRNA-seq; spatial transcriptomics; bulk transcriptomics; spatial immune phenotyping	POSTN+ CAF programs; CXCL13-related T-cell states; immune-excluded spatial patterns	Single-cell and spatial transcriptomics reveal POSTN cancer-associated fibroblasts correlated with immune suppression and tumor progression in non-small cell lung cancer (50); Cancer-associated fibroblasts drive CXCL13 production in activated T cells in non-small cell lung cancer (51).
Melanoma	Gut microbiome composition/function associates with anti–PD-1 response; responder-derived FMT can overcome resistance in a subset of PD-1–refractory patients and remodel host immunity	Shotgun metagenomics; host immune profiling/transcriptomics; clinical outcomes	Microbial diversity and taxa signatures; immune activation (e.g., IFN-γ–related programs)	Fecal microbiota transplant overcomes resistance to anti–PD-1 therapy in melanoma patients (52); Intestinal microbiota signatures of clinical response and immune-related adverse events in melanoma patients treated with anti-PD-1 (53).
RCC	A spatial architecture–embedding HLA signature integrates tumor–immune community structure and host HLA genetics to predict ICB response	Spatial profiling + transcriptomics; HLA genotyping; ML-derived signature	Spatial architecture–embedding HLA signature; coordinated CD8 T cell–macrophage patterns	A spatial architecture-embedding HLA signature to predict clinical response to immunotherapy in renal cell carcinoma (54).
HCC	Immune landscape–derived indices capture “immune-hot vs immune-cold/stemness-high” states and stratify immunotherapy response; ML-based immune indices link TIME features to outcomes	Bulk transcriptomics; immune landscape inference; ML integration	Immune Index/IMI-derived gene models; stemness/proliferation-enriched “cold” states	Immune index: A gene and cell prognostic signature for immunotherapy response in hepatocellular carcinoma (55).
Bladder cancer	Transcriptome-based characterization of variant histology supports predicting response to antibody–drug conjugates (ADC) and can guide precision treatment selection	Transcriptomics (expression profiling); clinicopathologic linkage	Transcriptomic subtypes/modules associated with ADC response	Abstract A029: Transcriptome analysis of variant histology bladder cancer reveals drug target heterogeneity (56); Abstract 4632: Lineage plasticity as a determinant of antibody-drug conjugate target expression in urothelial bladder cancer (57).
Colorectal cancer	AI-enabled digital pathology can predict MSI directly from H&E; MSI and transcriptomic subtypes reflect immune ecology linked to ICI benefit	Digital pathology + DL; transcriptomic subtyping (CMS)	MSI-H prediction from H&E; CMS immune/mesenchymal patterns	Deep learning can predict microsatellite instability directly from histology in gastrointestinal cancer (19).

ICI, immune checkpoint inhibitor; NSCLC, non-small cell lung cancer; RCC, renal cell carcinoma; HCC, hepatocellular carcinoma; CAF, cancer-associated fibroblast; TLS, tertiary lymphoid structure; MSI, microsatellite instability; CMS, consensus molecular subtype; ADC, antibody–drug conjugate; FMT, fecal microbiota transplantation.

## 3 AI-driven integration of multi-omics for immunotherapy biomarkers

**Figure 2:**
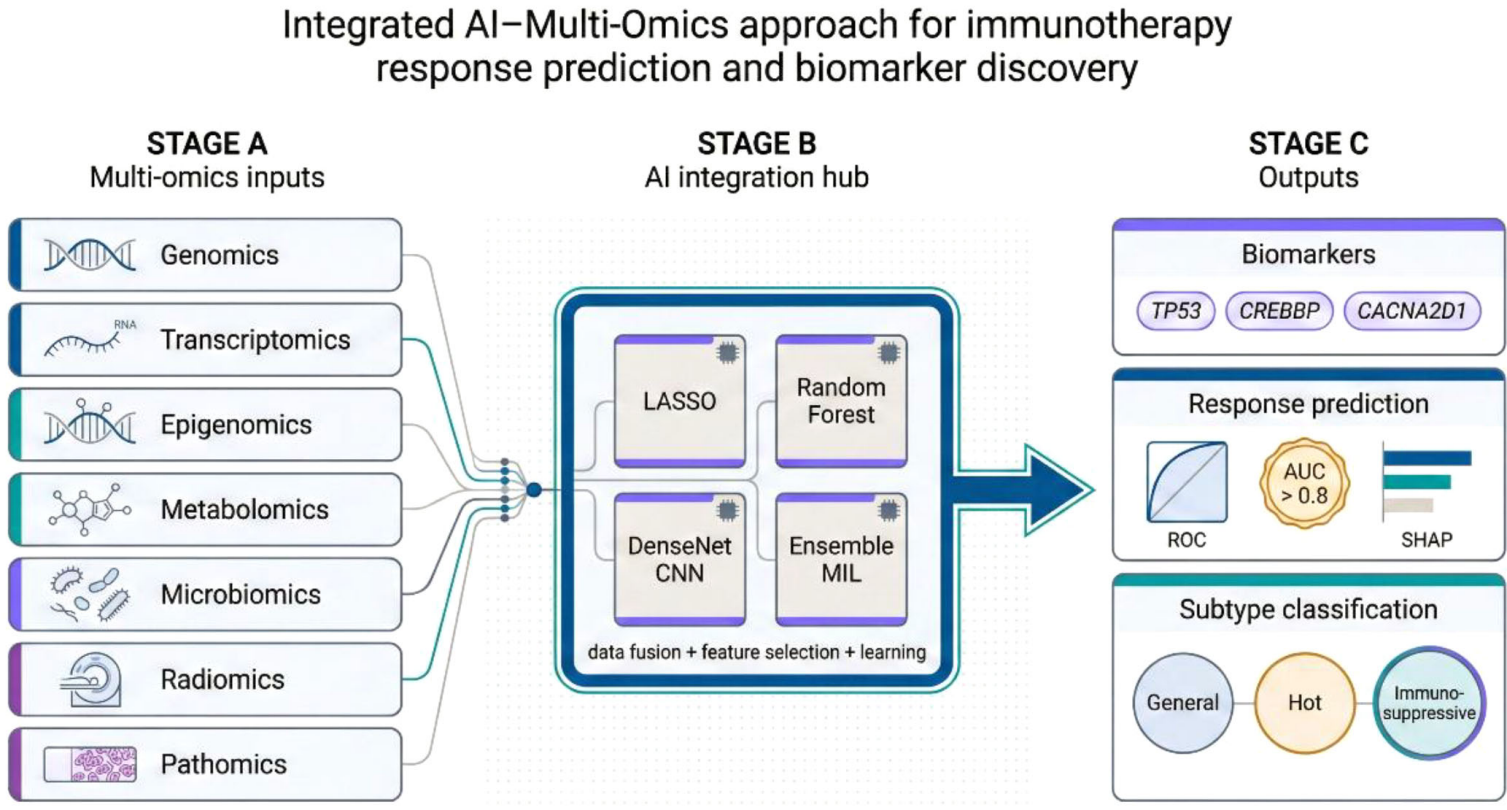
Integrated AI–multi-omics approach for immunotherapy response prediction and biomarker discovery. Three-stage workflow for building deployable predictors from heterogeneous omics. Stage A aggregates multi-omics inputs (genomics, transcriptomics, epigenomics, metabolomics, microbiomics, radiomics, pathomics). Stage B performs data fusion, feature selection, and model learning within an AI integration hub (e.g., LASSO, random forest, DenseNet CNN, ensemble multiple-instance learning). Stage C outputs clinically oriented products, including candidate biomarkers, probabilistic response prediction (ROC/AUC with model explainability such as SHAP), and biologically interpretable subtype classification (e.g., general, immune-hot, immunosuppressive).

Building on mechanistic insights into immune checkpoint inhibitor (ICI) efficacy and resistance, artificial intelligence (AI) can distill high-dimensional multi-omic data into clinically useful predictive tools (58, 59). By integrating genomic alterations, transcriptomic programs, immune and stromal context, imaging features, microbiome signals, and clinical variables, AI models detect interactions linked to immune sensitivity or resistance that are not captured by any single biomarker (24, 59) (**Figure 2**). As a result, composite predictors can outperform conventional markers such as PD-L1 staining or tumor mutational burden (TMB) (58, 59).

Representative studies highlight these gains (**Table 2**). In relapsed lymphoma, an artificial neural network integrating genomic status (including TP53/CREBBP), PD-L1 immunohistochemistry, and clinical covariates achieved an AUC of ~0.94 for predicting anti–PD-1 response, whereas individual factors were weak predictors (61, 66). In melanoma, multi-omic models combining mutational profiles with gene expression achieved validation AUCs of ~0.64–0.70, exceeding TMB alone, which was not significantly above random in that cohort (24, 25, 67). These results suggest that transcriptomic features, such as T cell–inflamed gene expression programs and interferon-γ–associated signatures, can be decisive even in tumors with high mutational load.

**Table 2:** Representative AI/ML models integrating multi-omics to predict immunotherapy outcomes.

Cancer type	Input modality	AI/ML model	Prediction target	Cohort size (as reported)	Study design	Performance (as reported)	References
GI cancers (CRC/STAD; multi-cohort)	H&E whole-slide histology	Deep residual learning (CNN)	MSI status (proxy for ICI benefit)	TCGA-STAD n=315; TCGA-CRC-DX n=360; TCGA-CRC-KR n=378; external DACHS n=378; external KCCH-GC n=185; (plus UCEC n=327 for generalization)	Retrospective + external validation	Patient-level AUC ~0.77–0.84 (CRC); AUC ~0.81 (STAD); external AUC ~0.84 (DACHS)	Deep learning can predict microsatellite instability directly from histology in gastrointestinal cancer (19).
Melanoma	Multi-omics tumor profiling (e.g., genomics + transcriptomics ± epigenomics/immune features)	Regularized regression (e.g., LASSO/elastic net)	ICB response/clinical benefit	Cohort size as reported in the study (checkpoint inhibitor–treated melanoma multi-omics cohort)	Observational, primarily retrospective	Multi-feature models identify resistance/response mechanisms and markers (study-reported metrics)	Integrative Tumor and Immune Cell Multi-omic Analyses Predict Response to Immune Checkpoint Blockade in Melanoma (60).
Relapsed/refractory DLBCL	Genomics + multiplex immunofluorescence/spatial proteomics + clinical variables	Artificial neural network (ANN)	Response to PD-1–containing regimens	Total n=50 (2018–2023; split as reported)	Retrospective	AUC ~0.97 (train), ~0.94 (test)	Multi-omics analysis and response prediction of PD-1 monoclonal antibody containing regimens in patients with relapsed/refractory diffuse large B-cell lymphoma (61)
NSCLC	CT radiomics (“habitat” features) ± molecular/clinical features	Deep learning radiomics (e.g., DenseNet-based)	Immunotherapy response/outcome prediction	Multi-institutional cohort n=246 (with validation as reported)	Retrospective	Study-reported response prediction performance (AUC/metrics per paper)	Integration of deep learning and habitat radiomics to predict immunotherapy response in non-small cell lung cancer (62).
NSCLC (microbiome)	Baseline shotgun metagenomics (gut microbiome)	Ecological topology–based scoring + ML	ICI efficacy prediction	Discovery cohort n=245 (plus validation cohorts as reported)	Retrospective, multi-cohort	Study-reported discrimination of responders vs non-responders	Custom scoring based on ecological topology of gut microbiota predicts immunotherapy efficacy in advanced non-small cell lung cancer (63).
LUAD	Transcriptomics ± other omics integration	Multi-omics ML risk modeling (e.g., CoxBoost + Elastic Net)	Prognosis/therapy stratification (immunotherapy-relevant)	TCGA-LUAD training n=502; external validation total n=544 (as reported)	Retrospective, multi-cohort	Stable performance across cohorts (C-index/metrics per paper)	Advancing lung adenocarcinoma prognosis and immunotherapy prediction via multi-omics integration and machine learning (64).
HNSCC	mRNA + lncRNA + miRNA + methylation + mutation	Multi-algorithm selection; final survival/response-linked model (e.g., StepCox + Ridge)	Immune subtype/immunotherapy sensitivity inference	TCGA-HNSCC + multiple GEO cohorts; additional immunotherapy cohorts (as reported)	Retrospective, multi-cohort	Best average C-index ~0.666 (as reported); immunotherapy cohorts used for external evaluation	Integrating immune multi-omics and machine learning to improve prognosis, immune landscape, and sensitivity to first- and second-line treatments for head and neck squamous cell carcinoma (65)

AI, artificial intelligence; ML, machine learning; DL, deep learning; CNN, convolutional neural network; MSI, microsatellite instability; ICI, immune checkpoint inhibitor; ICB, immune checkpoint blockade; H&E, hematoxylin and eosin; AUC, area under the receiver operating characteristic curve; C-index, concordance index; CRC, colorectal cancer; STAD, stomach adenocarcinoma; UCEC, uterine corpus endometrial carcinoma; DLBCL, diffuse large B-cell lymphoma; NSCLC, non-small cell lung cancer; LUAD, lung adenocarcinoma; HNSCC, head and neck squamous cell carcinoma; TCGA, The Cancer Genome Atlas; GEO, Gene Expression Omnibus.

AI methods for multi-omics broadly fall into (i) data integration and representation learning (e.g., unsupervised approaches) and (ii) supervised prediction (68, 69). On the integration side, algorithms address normalization and batch correction, dimensionality reduction, and feature selection across heterogeneous assays. Unsupervised strategies, including variational autoencoders and manifold learning, can fuse multiple omic layers into shared latent representations that compactly encode tumor state and reveal patient clusters with distinct immunogenomic profiles (68, 70). For example, consensus clustering of integrated head and neck cancer datasets uncovered three robust subtypes, immune-rich, metabolic, and stromal-differentiated, with markedly different ICI response rates (71, 72).

On the supervised side, regularized regression (e.g., LASSO), tree-based ensembles, and deep neural networks map multi-omic features to probabilistic response estimates. Outputs can be operationalized as predictive scores for patient stratification; in head and neck cancer, machine-learning–derived immunotherapy sensitivity indices immunotherapy sensitivity indices (e.g., CMPIS) separate likely responders, who may be candidates for ICI monotherapy, from likely non-responders who may require combination or alternative strategies (65, 73). These probabilistic scores can be used to enrich clinical trials, to prioritize combination regimens for predicted non-responders, and to support calibrated, patient-level estimates rather than categorical labels.

A further advantage of AI is extensibility to new modalities. Imaging-derived features can be integrated alongside molecular data: in non–small cell lung cancer (NSCLC), multimodal deep-learning models combining CT radiomics with genomic signatures produced attention heatmaps that corresponded to immune-cell–infiltrated regions on pathology, linking radiologic phenotype to immune context (59, 74). Spatial omics adds another layer; early AI efforts analyze multiplex immunohistochemistry or spatial transcriptomics to quantify immune-excluded versus inflamed architectures and to map suppressive niches at invasive margins (75, 76). AI can also leverage longitudinal sampling: models trained on serial liquid biopsies can detect early molecular signs of emerging resistance before clinical progression, enabling real-time monitoring (77).

Interpretability is increasingly prioritized to align predictions with biology and build clinical trust. Feature-importance rankings and SHAP-based attribution can highlight coherent drivers, for example, high CXCL9 expression together with favorable mutations such as PBRM1 in renal cancer (42, 78). Explainable modeling in melanoma has emphasized patient-specific neoantigen load weighted by MHC binding affinity as a leading predictor (79). In pathology-based models, gradient-based visualization techniques (e.g., Grad-CAM) has implicated tertiary lymphoid structure–like regions as influential signals, consistent with their established association with ICI benefit (80–82). Such alignment can also generate hypotheses when unexpected drivers (e.g., microbial metabolites) emerge as important features.

Overall performance reported for AI–multi-omics predictors is encouraging. Many studies describe validation AUCs in the ~0.70–0.85 range for discriminating responders from non-responders, improving on PD-L1 immunohistochemistry (often ~0.6 AUC) and on TMB (~0.6–0.7 across settings) (58, 59, 82). Integrated models also more often retain performance in independent cohorts, suggesting improved generalizability when multiple biological determinants are captured (23, 59). Importantly, multi-omics modeling reframes biomarker discovery: rather than a single yes/no marker, models yield multi-factor signatures or scores that can support treatment customization. In gastrointestinal cancers, an AI-derived signature incorporating 20 mutation features plus expression of three immune genes predicted response better than TMB and indicated that high-risk patients often carried MAPK alterations, suggesting benefit from adding MEK inhibition (83–85).

AI further enables immunotherapy-relevant tumor subtype classification. In melanoma, unsupervised learning identified a “hot tumor” subtype with high T cell signatures and low MYC activity that achieved superior ICI outcomes, whereas a MITF-low/pigmentation subtype with an invasive, de-differentiated phenotype was less responsive (86, 87). In NSCLC, integrated analyses stratified lung adenocarcinoma into subsets driven by EGFR signaling, inflammatory programs, or epithelial–mesenchymal transition; the high-plasticity/EMT subset was ICI-resistant but responsive to certain tyrosine kinase inhibitors, illustrating how integrated subtyping can guide sequencing and rational combinations (10, 88).

In sum, AI bridges complex multi-omic biology and clinical decision-making by synthesizing tumor–immune interaction signals into deployable predictive models and composite biomarkers, motivating continued work on clinical validation and translation.

## 4 Clinical applications and translational challenges

### 4.1 Predicting therapeutic outcomes and guiding combination therapies

A primary clinical use of AI–multi-omics is to predict which patients will benefit from immune checkpoint inhibitors (ICIs) (24, 25). Upfront identification of likely non-responders can spare patients the toxicity and cost of ineffective immunotherapy and allow timely transition to alternative modalities or trials (4). Conversely, confirming likely responders can facilitate rapid access to ICIs and, in selected contexts, support de-escalation from more toxic regimens (1). Importantly, AI models can also suggest mechanism-guided combination strategies by linking individualized tumor states to actionable vulnerabilities (24, 25).

In melanoma, an AI model integrating tumor exome and transcriptome data predicted response to anti–PD-1 therapy with validation performance around AUC ~0.7 (25). Retrospective analyses showed that patients assigned low response probabilities indeed had poorer outcomes (25). Notably, the model suggested that non-responders often retained high T-cell infiltration but exhibited an exhausted phenotype (25). This biologically nuanced stratification has informed combination strategies: a trial design emerged in which patients predicted to respond poorly despite high infiltration received anti–PD-1 plus either a LAG-3 inhibitor (targeting exhaustion biology) or added chemotherapy to immunogenically “reset” the tumor microenvironment (TME) (25, 67, 89). Early signals of improved outcomes in model-selected patients illustrate how AI prediction can be translated into rational combination regimens, rather than merely excluding patients from immunotherapy (25, 67).

In gastroesophageal cancers, where only a subset benefits from ICIs, an AI-based genomic signature has been developed to forecast survival benefit from PD-1 blockade (90, 91). The signature integrates mutation patterns, including co-occurrence such as TP53 and ARID1A alterations, with immune gene expression reflecting TME state (90). Patients designated high-risk by the signature had poor outcomes on immunotherapy alone, but the model also highlighted therapeutic options for combination (90). Specifically, many high-risk tumors displayed MAPK pathway activation, motivating addition of MEK inhibition (e.g., trametinib) to PD-1 blockade; preclinical testing supported synergy in signature-positive models (90–92). The signature further identified a subset with upregulated HSP90 stress proteins, generating a testable hypothesis that HSP90 inhibition could resensitize tumors to ICIs (90, 91). These examples underscore the clinical value of AI not only as a prognostic tool but also as a hypothesis-generating framework that connects multi-omic patterns to tractable combination strategies.

AI-informed combination approaches are also being explored in other cancers by targeting host and metabolic determinants of resistance. In non–small cell lung cancer (NSCLC), microbiome modulation through fecal microbiota transplantation (FMT) or probiotics is under investigation to enhance ICI efficacy (16, 17, 23). An AI model trained on metagenomic and clinical features identified bacterial species whose presence correlated with sustained responses; patients lacking these taxa were predicted to derive suboptimal benefit (16, 17, 23). A pilot trial subsequently tested donor FMT enriched for “favorable” bacteria such as Prevotella copri in predicted low-benefit patients receiving anti–PD-1 therapy (93). Some recipients showed increased T-cell activity and tumor shrinkage, providing early proof-of-concept for AI-guided microbiome adjuvants, while also highlighting the need for cautious donor selection and mechanistic monitoring (93).

Metabolic immunosuppression offers another translational axis. Multi-omics analyses of resistant tumors frequently show signatures of high lactate production and enriched lactylation marks, consistent with metabolic reprogramming that induces suppressive epigenetic states in immune cells (14, 15, 45). These findings have motivated trials combining checkpoint blockade with metabolic agents targeting lactate generation or export, such as lactate dehydrogenase inhibitors or monocarboxylate transporter blockade (14, 15, 45). The rationale is to reduce lactate-driven immune dysfunction, thereby converting immunologically “cold” tumors into more permissive, “hot” states (14, 45). Preclinical data suggest that lactate inhibition can restore dendritic cell function and improve anti–PD-1 efficacy, although definitive clinical results remain pending (14, 45).

Patient-specific multi-omic signatures are also being used to guide which modality to emphasize, not only whether to administer ICIs. In head and neck squamous cell carcinoma (HNSCC), an ML-refined signature (CMPIS) combining immune and stemness indicators stratified tumors into immune-hot versus immune-cold states (94). Low SPI scores reflected inflamed, immunogenic tumors, whereas high scores indicated suppressive, less differentiated TMEs (94). Retrospective analyses suggested that low-SPI patients responded well to ICIs (often with conventional chemotherapy), whereas high-SPI patients derived greater benefit from EGFR-targeted strategies than from immunotherapy (49, 94). Such signatures are proposed to inform first-line treatment allocation, aiming to maximize efficacy by matching tumor state to modality.

In hepatocellular carcinoma (HCC), where ICIs are now integrated into standard therapy (e.g., anti–PD-L1 plus anti-VEGF), AI models combining tumor genomics with blood biomarkers are being explored to identify candidates for conversion therapy, a systemic treatment intended to downstage unresectable disease into resectability (27, 28, 58). An AI radiomics model predicted which patients would achieve substantial downstaging on immunotherapy (27, 28). Predicted responders could be routed to intensified protocols combining ICIs with locoregional interventions such as transarterial chemoembolization, enabling surgical resection in a meaningful fraction; predicted non-responders could be spared ineffective ICI exposure and offered alternative trials (27, 28). This illustrates model-guided escalation and redirection based on individualized response probabilities.

Neoadjuvant immunotherapy represents another clinically relevant setting because response can be quantified at surgery. In resectable NSCLC, only a subset achieves major pathological response (MPR) or pathologic complete response (pCR) after neoadjuvant ICIs (95, 96). Multi-omics models integrating tumor genomics, baseline imaging, and peripheral immune markers have been developed to predict MPR/pCR (58, 59, 74). In a trial of neoadjuvant camrelizumab plus chemotherapy, biomarker analyses reportedly identified factors associated with a large proportion of pCR cases (95). If prospectively validated, these tools could triage patients toward neoadjuvant immunotherapy versus alternative preoperative strategies, potentially reducing overtreatment and improving surgical outcomes.

### 4.2 AI integration into pathology and imaging workflows

Alongside systemic decision-making, AI is being embedded into routine diagnostic pipelines to support immunotherapy decisions and monitoring. Digital pathology platforms use deep learning to quantify PD-L1 expression and tumor-infiltrating lymphocyte (TIL) densities, improving reproducibility over manual reads and enabling spatial descriptors such as TIL clustering at invasive margins (82, 97, 98). These metrics can complement molecular profiling by providing rapid, standardized immune-context measurements from routine biopsies.

Radiology is similarly adopting AI-derived imaging biomarkers. Radiomic texture and heterogeneity measures from CT, MRI, and PET can capture tumor architecture and stromal features associated with immune infiltration or exclusion (21, 59). AI analysis of baseline CT scans in lung cancer has been reported to predict response and survival after ICIs independently of clinical factors, using features such as enhancement heterogeneity, tumor shape, and tumor–tissue interface characteristics that correlate with immune stroma (99–101). In practice, pathology-derived AI immune metrics and radiomic scores could be jointly considered to guide immunotherapy versus alternative options.

Ultrasound, including contrast-enhanced ultrasound, is also being explored for serial assessment. Ultrasound imaging may quantify therapy-induced changes in vascularity and perfusion that accompany immune infiltration, potentially enabling on-treatment evaluation even when size criteria lag (102, 103). Although still experimental, such approaches could help detect immune-related inflammation patterns, distinguish true progression from pseudoprogression, and prompt timely adjustments to treatment.

### 4.3 Real-world evidence and multicenter validation

Robust generalization across institutions, platforms, and patient demographics is essential for clinical use. Multicenter validation and real-world evidence are therefore central, and several examples illustrate both promise and remaining risks. An international validation of a deep-learning radiomic model for NSCLC, trained to predict anti–PD-1 response from pretreatment CT scans combined with blood inflammatory markers, maintained AUCs of ~0.82–0.86 across multiple hospitals in Europe and China (104, 105). Model outputs correlated with overall survival and are being evaluated prospectively, supporting the feasibility of cross-region transport when predictors capture durable biological correlates (104, 105).

In diffuse large B-cell lymphoma, an artificial neural network integrating mutation status, PD-L1 expression, and clinical factors achieved ~0.94 AUC in initial cohorts and retained similar performance in independent multicenter sets (61, 66). Importantly, predicted response scores correlated with spatial immune profiling: responders showed higher densities of PD-1^+^ T cells proximal to PD-L1^+^ macrophages, consistent with active immune engagement, whereas non-responders exhibited diffuse suppression (61). Such convergence between AI predictions and spatial biology strengthens mechanistic plausibility and external validity.

In gastrointestinal cancers, multi-omics signatures derived in one cohort have been tested in independent datasets and public repositories such as TCGA (106). A prognostic immunogenomic signature for microsatellite-stable colorectal cancer validated in TCGA outperformed tumor mutational burden and PD-L1 status in predicting survival on immunotherapy (91, 107). Combined DNA/RNA classifiers for gastric cancer also retained high sensitivity and specificity in independent Asian cohorts, underscoring cross-population validity amid regional genetic and environmental variation (108, 109).

Retrospective analyses further support reproducibility of some AI-derived signals. In melanoma, an “immune responsiveness” score applied to pooled transcriptomic datasets stratified benefit across studies and recapitulated expected biology: higher interferon-γ and CXCL9 expression among predicted responders and enrichment of MYC targets and WNT/β-catenin signaling among predicted non-responders (110, 111). In lung adenocarcinoma, a stemness-based classifier separating immune-hot versus immune-cold tumors was validated across seven cohorts totaling over a thousand patients; pathology review confirmed paucity of CD8^+^ T cells in high-stemness tumors by immunofluorescence (49, 112).

Prospective practice data are emerging as well. In bladder cancer, a model integrating urine tumor DNA mutations and methylation markers has been prospectively tested across centers, reducing invasive cystoscopy by flagging patients likely stable on immunotherapy versus those likely progressing (113, 114). Meta-analytic work suggests that single biomarkers often have lower generalizability in diverse populations, reinforcing the need for multi-omic composites that better capture heterogeneity across demographics and environments (115, 116). Analyses similarly show that trials incorporating AI or multi-omics immunotherapy biomarkers have increased substantially since 2020, with many specifying validation and biomarker-guided therapy endpoints (117, 118). Nonetheless, even strong models can drift with new data distributions (sequencing platforms, preprocessing pipelines, or imaging protocols), and early deployment studies indicate that performance may drop until harmonization and recalibration are applied (119).

### 4.4 Challenges in implementation

Despite encouraging evidence, broad adoption faces major practical and ethical barriers. Data heterogeneity and standardization remain central: different centers generate omics and imaging data with distinct platforms, protocols, and recording formats, creating batch effects that compromise portability (68, 69). Harmonized pipelines and quality standards are required, and model recalibration may be necessary when deployed across scanners or sequencing workflows.

Privacy and governance constraints further limit access to the diverse cohorts needed for robust training (120). Federated learning is emerging as a solution by enabling collaborative training without moving patient-level data (121, 122). Federated efforts, such as those combining data from multiple hospitals, have achieved performance comparable to pooled-data training, illustrating feasibility while addressing privacy and heterogeneity (123, 124). Still, federated approaches require consistent annotation, secure aggregation, and rigorous governance to ensure data quality and accountability.

Prospective validation remains limited: most predictors are retrospective, and clinical translation requires evidence that acting on predictions improves outcomes (6, 125). Prospective biomarker-guided trials are underway in settings such as neoadjuvant lung cancer, but larger randomized studies are needed before routine adoption. Regulatory approval also demands demonstration of analytical validity, clinical validity, and clinical utility, as well as cybersecurity and post-market surveillance; adaptive models raise additional issues of versioning, re-approval, and drift monitoring (126, 127).

Interpretability and clinician acceptance are pivotal, as many deep learning models are perceived as black boxes. Explainable AI methods (e.g., SHAP and Grad-CAM) can clarify drivers of predictions, and visualization of multi-omic correlations can make model reasoning more tangible (128, 129). Another translation strategy is distillation into parsimonious, assayable signatures, for example, reducing complex models to small gene panels or immunohistochemistry/PCR surrogates compatible with standard workflows (58).

Equity considerations are paramount. Models trained on predominantly European-ancestry or region-limited cohorts may underperform in underrepresented groups, potentially worsening disparities (130, 131). Mitigation requires diverse cohort inclusion, fairness-aware training and calibration, subgroup-stratified reporting, and ongoing surveillance for systematic underperformance. Workflow integration is also non-trivial: outputs must be presented in clinician-friendly interfaces (e.g., dashboards showing risk spectra, confidence intervals, and key explanatory factors) and embedded within electronic records to avoid additional cognitive burden (132–134).

**Figure 3:**
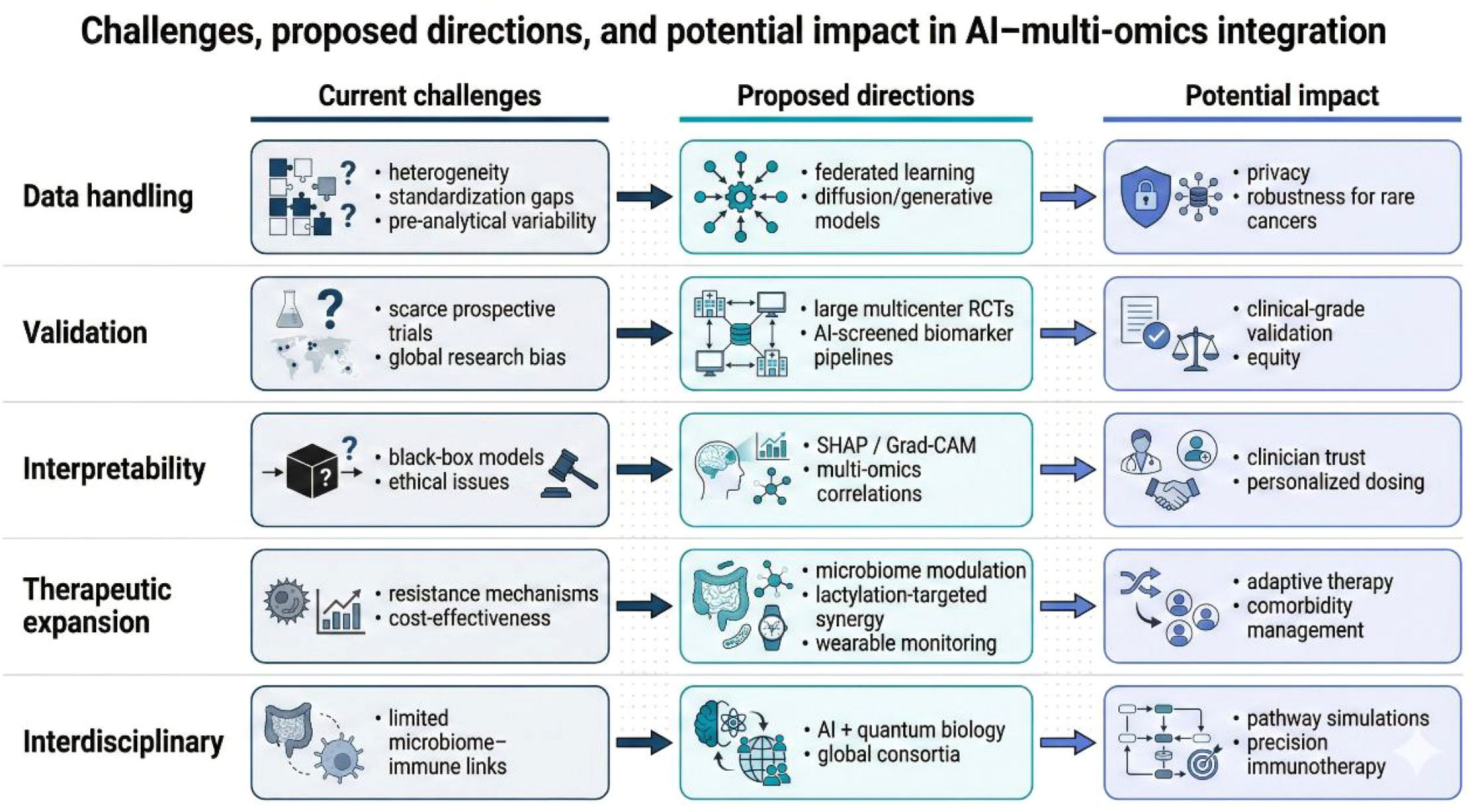
Challenges, proposed directions, and potential impact in AI–multi-omics integration. Structured “challenge → direction → impact” framework across five dimensions: (i) data handling (heterogeneity/standardization/pre-analytical variability → federated learning, diffusion/generative models → privacy and robustness for rare cancers), (ii) validation (scarce prospective trials/global research bias → large multicenter RCTs, AI-screened biomarker pipelines → clinical-grade validation and equity), (iii) interpretability (black-box/ethical issues → SHAP/Grad-CAM and multi-omics correlation analyses → clinician trust and personalized dosing), (iv) therapeutic expansion (resistance mechanisms/cost-effectiveness → microbiome modulation, lactylation-targeted synergy, wearable monitoring → adaptive therapy and comorbidity management), and (v) interdisciplinary integration (limited microbiome–immune links → AI + quantum biology, global consortia → pathway simulations and precision immunotherapy).

Finally, cost-effectiveness will shape uptake. Multi-omics profiling and advanced analytics can be expensive and unevenly available, and reimbursement will require evidence of value. Early health-economic analyses suggest that if an AI test can spare even ~20% of patients from futile therapy, it may be cost-saving, but real-world confirmation is needed (135, 136). Looking forward, translational roadmaps emphasize harmonized data handling, federated learning, explainable modeling, prospective trials, and expansion to adjunct strategies such as microbiome and metabolic modulation, with emerging concepts including wearable AI monitoring for toxicity and exploratory computational approaches (e.g., quantum computing) for complex simulations (**Figure 3**).

**Figure 4:**
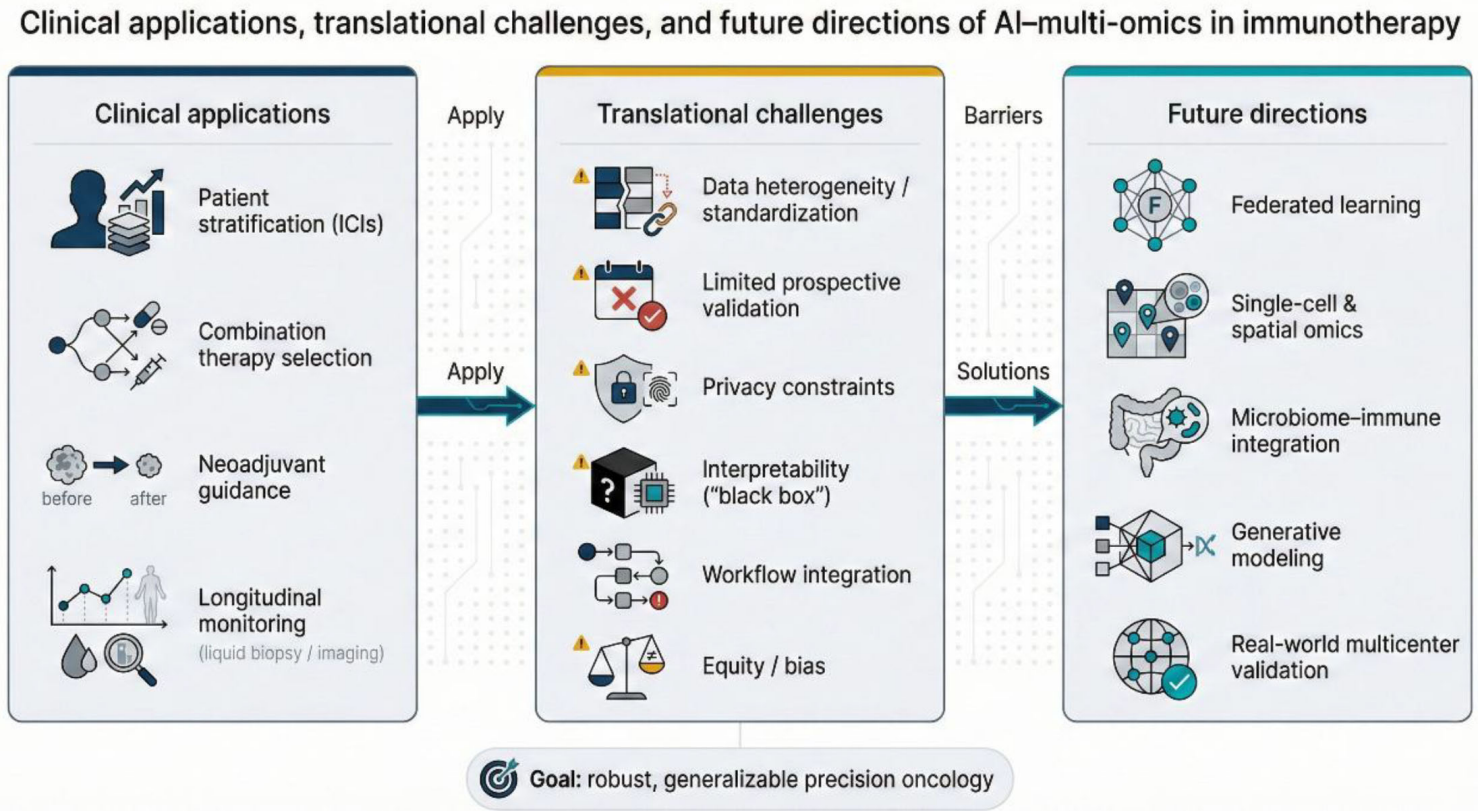
Clinical applications, translational challenges, and future directions of AI–multi-omics in immunotherapy. Conceptual map linking real-world clinical use cases to key barriers and solution pathways. Clinical applications include patient stratification for ICIs, combination-therapy selection, neoadjuvant guidance, and longitudinal monitoring (liquid biopsy/imaging). Translational challenges include data heterogeneity/standardization, limited prospective validation, privacy constraints, interpretability (“black box”), workflow integration, and equity/bias. Future directions highlight solution-oriented strategies such as federated learning, single-cell & spatial omics, microbiome–immune integration, generative modeling, and real-world multicenter validation, aiming for robust and generalizable precision oncology.

In summary, AI–multi-omics tools are increasingly positioned to personalize ICI use by predicting benefit, guiding combinations, enabling neoadjuvant triage, and supporting monitoring via pathology and imaging (**Figure 4**). Achieving routine clinical adoption will depend on multicenter validation, prospective evidence of utility, standardization and privacy-preserving infrastructure, interpretable and equitable model development, seamless integration into workflows, and demonstrable clinical and economic value.

## 5 Future directions and emerging technologies

### 5.1 Advanced AI–multi-omics paradigms

A major frontier is integrating single-cell and spatial omics data with AI. Traditional bulk sequencing averages signals across millions of cells, potentially obscuring critical intratumoral and microenvironmental heterogeneity. Single-cell RNA-seq, ATAC-seq, and multimodal single-cell assays that jointly measure gene expression and cell-surface proteins can delineate the multitude of cell types and functional states within the tumor and tumor microenvironment (TME) (86, 137). Applying AI to these high-dimensional datasets can identify subtle subpopulations or gene programs associated with immune escape. In NSCLC, for example, a multi-omic single-cell study confirmed that a rare SOX2^+^ progenitor population drives lineage plasticity and ICI resistance (10). Researchers are therefore developing deep learning models that predict therapy outcome from the cellular ecosystem defined by single-cell data, learning which cellular composition patterns, such as regulatory-to-cytotoxic T-cell balance or the presence of specific myeloid suppressor subsets, track with response.

The scale of single-cell and spatial data, often thousands of cells or spatial spots per patient, demands architectures that can model relationships among cells. Graph neural networks represent cells as nodes in an interaction graph, capturing neighborhood structure and cell–cell communication (138, 139). Transformer models, which excel at long-context representation, can treat cells (or spatial spots) as elements in a sequence and infer higher-order dependencies across the TME (140). These approaches enable models to learn patterns such as adjacency of PD-L1–expressing tumor cells to exhausted T cells, or spatial clustering of suppressive myeloid states, to inform response prediction (138, 140). A forward-looking vision is a clinically integrated system that ingests a spatially resolved tumor map and outputs both a response probability and an interpretable annotation highlighting immunosuppressive niches and immune-active regions, supporting pathologists and oncologists in near real time.

Federated and distributed learning paradigms are also advancing. By enabling institutions to train models locally and share only parameter updates, federated learning can facilitate multi-center model development without pooling raw data (121, 141). These frameworks are becoming more sophisticated through secure multi-party computation and, in some proposals, blockchain-based audit trails for transparency and traceability. In immunotherapy, federated networks could allow large cancer centers to contribute to a continuously learning predictor that improves as outcomes accrue across sites (122). Supporting feasibility, the Oncology Federated Learning Network reported training a metastatic cancer survival prediction model across 17 institutions without pooling data, achieving performance within 2% of a centralized model (121, 122, 124). Extending this paradigm to ICI response could accelerate improvement and improve generalizability across populations. Parallel efforts in privacy-preserving AI, such as homomorphic encryption, which enables computation on encrypted data, may further reduce barriers to incorporating electronic health records and radiology archives into training and validation (122, 123).

Generative AI represents another expanding toolkit. GANs and diffusion models can synthesize realistic multi-omics profiles that follow statistical patterns of real cohorts, augmenting training sets when data are scarce (142, 143). This is especially relevant for rare cancers and underrepresented patient groups, where limited sample size increases risk of overfitting and biased performance. Generative models can also support mechanistic exploration by simulating multi-omics “what-if” scenarios: given a tumor’s genomic context, they can generate plausible transcriptomic and immune-cell profiles consistent with either response or resistance, suggesting molecular shifts that might convert non-responders to responders (143). In parallel, foundation-model strategies are emerging, including large language–model–like approaches for biomedical data: models pre-trained on large corpora of tumor transcriptomes or clinical narratives may be fine-tuned with limited task-specific data to predict immunotherapy outcomes more robustly (144, 145).

On the computational horizon, quantum computing is being explored for its potential to handle the combinatorial complexity inherent to multi-omics. Quantum algorithms could, in principle, accelerate feature selection or simulate molecular interactions within the TME beyond classical brute-force approaches (146, 147). While practical quantum computing for clinical medicine remains years away, quantum-inspired algorithms for optimization are already being investigated, aiming to improve parameter search and enable exploration of extremely high-dimensional interaction spaces that are otherwise computationally prohibitive.

Emerging cross-disciplinary fields further broaden the scope of prediction. Cardio-oncology exemplifies the move from efficacy-only prediction toward joint optimization for benefit and harm: AI is being used to anticipate immune-related cardiac adverse events, including ICI-associated myocarditis, by integrating cardiac biomarkers and imaging with oncologic variables (148, 149). The American Heart Association has issued statements emphasizing opportunities for AI and multi-omics to forecast long-term immunotherapy risks, including cardiotoxicity (150). Such work suggests future predictors may concurrently estimate likelihood of tumor control and probability of severe adverse events, tailoring therapy to maximize benefit while minimizing harm.

### 5.2 Personalized medicine and biomarker validation

Clinical translation will require rigorous validation and refinement toward truly personalized immunotherapy, with emphasis on actionable biomarkers that drive decisions rather than merely correlating with outcomes. Accordingly, prospective trials increasingly incorporate multi-omics and AI analyses as exploratory, and sometimes primary, endpoints, creating the evidentiary pathway for clinical deployment.

Liquid biopsy is a leading avenue for personalization because it can be obtained serially, enabling longitudinal monitoring of tumor evolution and immune engagement. AI models integrating time-resolved circulating tumor DNA (ctDNA), circulating immune-cell features, and related blood-based analytes are being tested to predict outcomes and detect resistance early (29, 35, 113). In bladder cancer, a liquid-biopsy methylation signature has been used to guide therapy adaptation: patients whose blood shows a “cold tumor” methylation pattern after several weeks on ICIs may be escalated to combination therapy, whereas those with a “hot tumor” pattern may continue ICIs alone (30, 113). In melanoma, early declines in ctDNA during therapy correlate with response; models combining ctDNA dynamics with radiographic changes and on-treatment transcriptomic shifts can improve prediction beyond any single modality (25, 151). Such adaptive, biomarker-driven strategies embody the personalized medicine ideal by adjusting treatment in near real time based on each patient’s evolving molecular data.

Translation to routine care often demands simplification. Whole-exome sequencing and RNA-seq remain variably available and may not meet turnaround constraints in all clinics. A practical strategy is to develop targeted panels that capture the most informative features discovered by AI, for example, a defined set of expression markers, a curated mutation list, and selected microbiome taxa, so that a focused assay can approximate the predictive signal of broader profiling (29, 152). AI can also prioritize biomarkers likely to remain robust when implemented with simpler measurement technologies such as PCR or immunohistochemistry (113). In NPC, where EBV-driven enhancers activate CACNA2D1, an enhancer activation score based on a small set of epigenetic marks has been proposed as a biomarker that could be measured by a targeted assay and used to select patients for enhancer-blocking epigenetic strategies alongside ICIs (12, 13).

Prospective trials that explicitly test AI-guided treatment adaptation represent a critical next step. “Adaptive immunotherapy” designs envision models that continuously analyze patient data during therapy and recommend modifications, such as adding an agent, switching regimens, or adjusting intensity, when predicted response probability drops below a threshold (28, 113). Operationalizing such trials requires rapid turnaround of complex assays, real-time computation, and an integrated clinical decision engine, but successful execution could shift immunotherapy management from static, one-size-fits-all regimens to dynamic, data-informed control policies (153, 154).

Wearable and digital biomarkers are likely to expand monitoring further. Physiologic signals from wearables (activity, sleep, heart-rate variability) may provide early indicators of toxicity and possibly efficacy; for example, fatigue patterns could reflect cytokine-mediated immune activation (155, 156). AI can integrate these continuous streams with molecular and imaging features to produce a holistic patient-status estimate and alert care teams to impending immune-related adverse events for preemptive intervention (75). Proof-of-concept work has predicted severe immunotherapy toxicity by combining baseline cytokine profiles with T-cell receptor repertoire features, suggesting that periodic molecular monitoring may be complemented by continuous digital signals (157). Looking ahead, generative AI may also support “digital twin” concepts, simulating likely trajectories under alternative regimens to aid selection of safer and more effective options for individual patients.

Interdisciplinary integration remains essential. Microbiome–immune axis modeling links microbial metabolites to immune-cell states in the TME, suggesting microbiome modulation strategies to overcome resistance (158). Integration with metabolic and epigenetic research is similarly influential; discoveries such as lactylation emerged from cross-talk between metabolism and immunology, and AI is helping map where metabolic interventions might integrate with immunotherapy regimens (14, 15). Systems biology and network-science approaches, sometimes framed as immune-atlas initiatives, may further enable simulation of how perturbations (e.g., blocking TGF-β) propagate through tumor–immune dynamics, with AI serving as the computational engine for such high-dimensional simulations (159, 160).

Global research efforts are coalescing around these directions. Initiatives such as the National Cancer Institute’s Human Tumor Atlas Network and international consortia on immunotherapy biomarkers are generating rich datasets that AI can leverage. Concurrently, a growing emphasis on open data and open models is enabling external validation and community scrutiny, reducing the risk that overfitting or bias goes unnoticed. In conclusion, the coming years are poised to deliver deeper mechanistic insight and more powerful predictive tools for immunotherapy as AI advances alongside experimental technologies.
